# Case report: Aumolertinib plus gumarontinib in a patient with EGFR mutated non-small-cell lung cancer harboring acquired MET amplification following progression on afatinib plus crizotinib

**DOI:** 10.3389/fphar.2025.1525251

**Published:** 2025-02-14

**Authors:** Jia-Jun Hui, Sheng-Jun Ding, Bao-Dong Qin, Ning Ding

**Affiliations:** ^1^ Department of Medical Oncology, Wuxi Huishan District People’s Hospital, Affiliated Huishan Hospital of Xinglin College, Nantong University, Wuxi, China; ^2^ Department of Medical Oncology, Shanghai Changzheng Hospital, Naval Medical University, Shanghai, China

**Keywords:** non-small cell lung cancer, EGFR mutation, MET amplification, aumolertinib, gumarontinib

## Abstract

**Background:**

Although it remained fully unclear about the optimal regimen for Epidermal growth factor receptor tyrosine kinase inhibitor (EGFR-TKI) resistant non-small cell lung cancer (NSCLC) patients with Mesenchymal-Epithelial Transition factor (MET) amplification, the dual inhibition of EGFR inhibitor with MET inhibitor were attempted in clinical practice. There is very limited literature on the subsequent treatment when patients develop the resistance to this combination regimen.

**Case summary:**

The patient, a 49-year-old female, initially presented with EGFR exon 21 L858R metastatic lung adenocarcinoma, treated successfully with first-line afatinib on September 2022 with a progression-free survival (PFS) of 8.0 months. On May 2023, she developed chest tightness and was found to have pericardial and pleural effusions containing malignant cells, indicating disease progression. Next-generation sequencing using pericardial effusion revealed concurrent EGFR L858R mutation and MET amplification. Then, afatinib plus crizotinib was initiated as second-line regimen, achieving stable disease with a PFS of 13.5 months. On July 2024, the patient developed the resistance to afatinib plus crizotinib due to the appearance of brain metastases. Then, this patient was administrated with aumolertinib plus gumarontinib as third-line regimen. Remarkably, this led to significant radiographic improvement of brain metastases. This patient is still undergoing third-line treatment, with a PFS of 3.7 months.

**Conclusion:**

This case underscores the importance of re-challenge using third-generation EGFR-TKI with novel MET-TKI after the failure of second-generation EGFR-TKI plus crizotinib in EGFR-TKI resistant NSCLC patients with MET amplification, especially in patients with brain metastases. The successful application of aumolertinib plus gumarontinib highlights its potential in overcoming MET amplification-induced EGFR-TKI resistance, which warrants further investigation in future large-scale clinical trials.

## Background

Epidermal growth factor receptor tyrosine kinase inhibitors (EGFR-TKIs) represent the standard therapeutic modality for patients with advanced non-small cell lung cancer (NSCLC) harboring EGFR mutations (Soria et al., 2018; Hsu et al., 2018). Nevertheless, almost all patients eventually experience acquired resistance to EGFR-TKI (Qin et al., 2024). The approved EGFR-TKIs are classified into first-, second-, and third-generation inhibitors based on their mechanisms of action and target specificity (Singh et al., 2023). First-generation EGFR-TKIs, such as gefitinib and erlotinib, are reversible inhibitors. Second-generation EGFR-TKIs, including afatinib and dacomitinib, are irreversible inhibitors that covalently bind to EGFR. However, resistance to first- or second-generation EGFR-TKIs frequently developed due to the acquired T790M mutation, which occurs in approximately 50%–60% of NSCLC patients treating with first- or second-generation EGFR-TKIs (Westover et al., 2018). The emergence of T790M mutation after first- or second-generation EGFR-TKIs has promoted the development of third-generation EGFR-TKI, such as osimertinib and aumolertinib. Third-generation EGFR-TKIs were designed to irreversibly and covalently bind to Cys797 residue of EGFR while retaining the function to inhibit EGFR activity even with EGFR T790M mutation. Despite the significant advancements achieved with third-generation EGFR-TKIs, however, resistance to third-generation EGFR-TKIs would develop inevitably over the course of treatment, similar to first-, and second-generation EGFR-TKIs (Blaquier et al., 2023).

After the failure of EGFR-TKI treatment, a thorough testing for acquired resistance mechanisms is recommended to guide subsequent treatment. Among these acquired resistance mechanisms, MET amplification is the prevalent mechanism driving resistance to EGFR-TKI, with a reported incidence ranged from 10% to 66% (Wu et al., 2024). MET amplification could be observed more frequently with the third-generation EGFR TKI than first- or second-generation EGFR-TKIs, especially in first-line setting for previously untreated EGFR-mutated NSCLC (Westover et al., 2018). Following the failure of EGFR TKIs, platinum-based chemotherapy remains the standard therapeutic option, although immunotherapy combination represents a promising therapeutic strategy for these patients. As for EGFR-mutated NSCLC patients who develop MET amplification after disease progression on EGFR TKIs, the dual inhibition of MET and EGFR may provide clinical benefit. Previously, crizotinib in combination with EGFR-TKI was identified as a potential modality for these patients (Wu et al., 2024). Subsequently, novel selective MET inhibitors have also been attempted. For example, INSIGHT2 trial reported that tepotinib plus osimertinib showed promising activity and acceptable safety in EGFR-mutated NSCLC with MET amplification as a mechanism of resistance to first-line osimertinib, suggesting a potential chemotherapy-sparing oral targeted therapy option for these patients (Wu et al., 2024). Currently, a range of highly-selective MET inhibitors (gumarontinib, savolitinib, etc.) and EGFR inhibitors (aumolertinib, furmonertinib, etc.) are commonly used in clinical practice (Chu et al., 2022). Do different combinations of these agents have the potential to effectively overcome EGFR-TKI resistance? In addition, it remains unclear on whether the novel MET inhibitor could overcome crizotinib resistance among these patients. Here, we reported an EGFR-mutated NSCLC patients with MET amplification after afatinib treatment who obtain significant clinical benefit from the combination of aumolertinib with gumarontinib after failure on afatinib plus crizotinib.

## Case presentation

A 49-year-old female patient with no history of smoking, no family history of malignancies, and no underlying comorbidities presented with a right-sided cervical mass. A chest enhanced CT scan conducted on 20 August 2022, showed a mass in the upper lobe of left lung accompanied by obstructive pneumonia, with multiple lymph node metastases involving the bilateral supraclavicular regions, bilateral pulmonary hila as well as the mediastinum. Metastasis to the apical-posterior segment of the left upper lobe was also observed. Brain MRI, bone scintigraphy and other imaging examination showed no signs of metastases in the central nerve system, the skeletal system or other organs.

**FIGURE 1:**
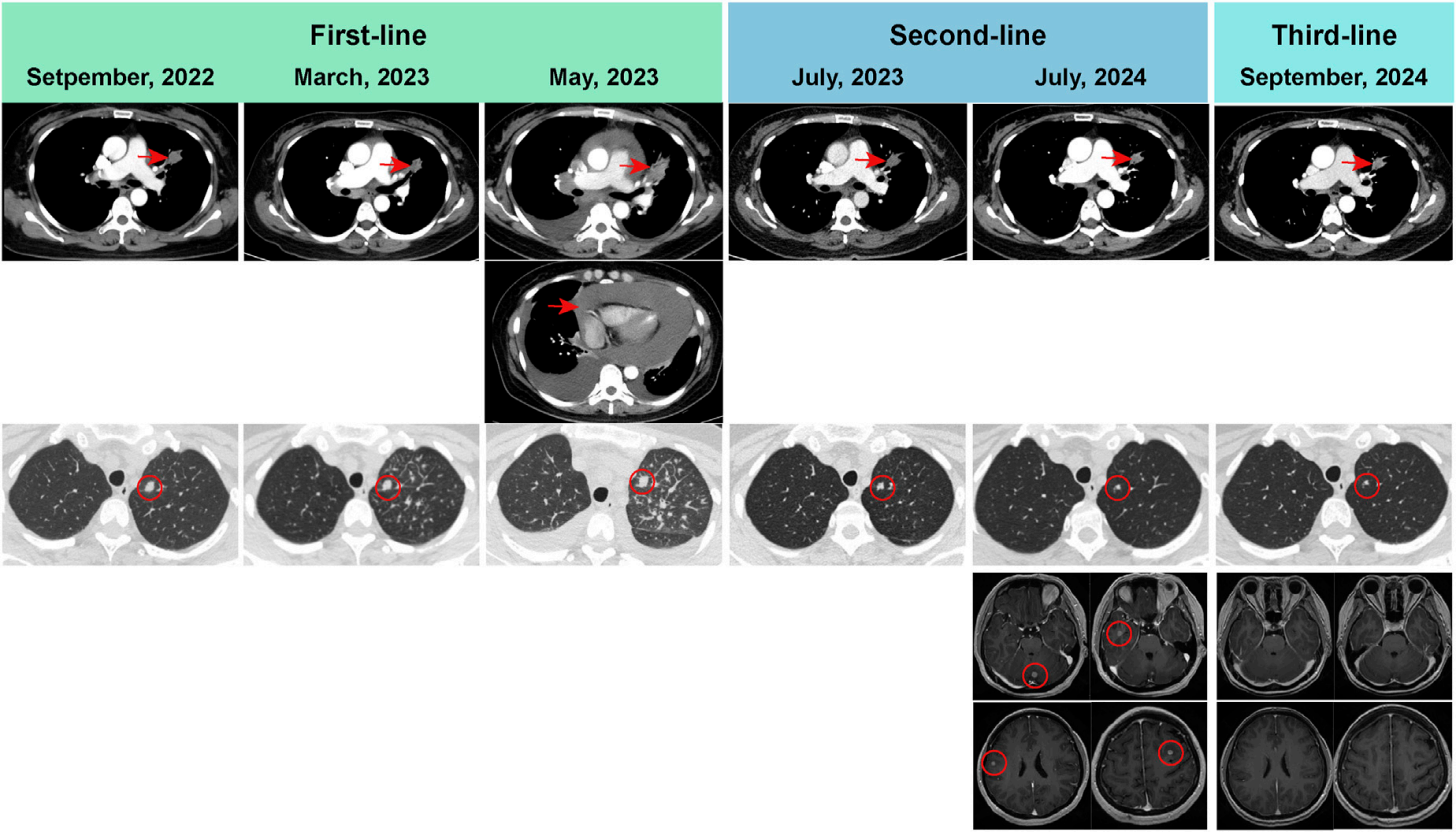
Radiological evaluation of primary lung lesion, as well as brain metastatic lesions. Progression disease was developed, with a PFS of 7.8 months on first-line afatinib treatment (afatinib 30 mg once daily). Next-generation sequencing on the pericardial effusion showed EGFR L858R mutation with MET amplification. The regimen consisted of afatinib (30 mg oral once daily) and crizotinib (250 mg oral once daily) was used as second-line regimen. The patient developed brain metastases, with a PFS of 13.5 months on second-line therapy. Aumolertinib (110 mg oral once daily) and gumarontinib (300 mg oral once daily) was used as third-line regimen. The radiological evaluation showed that the brain metastasis was disappeared. This patient is currently continuing follow-up and treatment on aumolertinib and gumarontinib, with a PFS of 3.7 months. PFS, progression-free survival.

**FIGURE 2:**
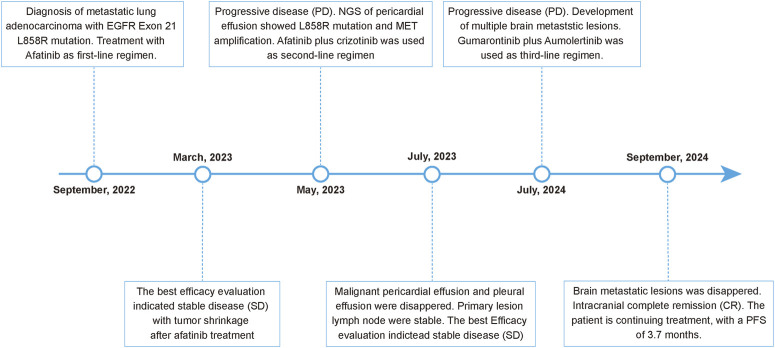
Timeline of the patient’s clinical course and therapeutic regimens.

On 29 August 2022, the patient underwent a supraclavicular lymph node fine-needle aspiration biopsy. Poorly differentiated adenocarcinoma was confirmed by the pathological findings, and immunohistochemistry displayed CK7 (+), TTF-1 (+), NapsinA (+), Ki-67 (+, 30%), P40 (−), CK5/6 (−), Syn (−), CD56 (−), CgA (−), PD-L1 (−). Next-generation sequencing analysis was performed using capture-based targeted sequencing across a panel of 520 genes (Burning Rock Biotech, Guangzhou, China) on tumor tissues and plasma to identify genomic alterations, which identified EGFR exon 21 L858R mutation (mutation allele frequency in tissue: 44.7%), CDK12 amplification (copy number in tissue: 21.4), ERBB2 amplification (copy number in tissue: 24.6) as well as a TMB of 7.89 mutations/Mb, with microsatellite stability (MSS) (Table 1). The patient was administrated orally with afatinib 30 mg once daily on 14 September 2022. After afatinib treatment, the follow-up chest CT scan showed a reduction in the left upper lobe mass, decreased obstructive inflammation, with the shrinkage of lymph node in the bilateral supraclavicular, bilateral hilar, and mediastinal regions. The best efficacy evaluation indicated stable disease (SD) with tumor shrinkage after afatinib treatment (Figures 1, 2).

**TABLE 1:** Genetic alterations detection results (tier I and tier II alteration) from twice NGS analysis.

	First NGS analysis at 2022-09-01		Second NGS analysis at 2023-05-10	
Sample	Tissue	Plasma	Pericardial effusion	Plasma
EGFR	EGFR L858R (MAF = 44.7%)	EGFR L858R (MAF = 7.28%)	EGFR L858R (MAF = 30.2%)	EGFR L858R (MAF = 3.5%)
MET			MET amplification (CN = 4.8)	
TP53	TP53 Exon 4 splicing mutation (MAF = 54.9%)	TP53 Exon 4 splicing mutation (MAF = 7.16%)		
ERBB2	ERBB2 amplification (CN = 24.6)	ERBB2 amplification (CN = 4.0)	ERBB2 amplification (CN = 11)	
CDK12	CDK12 amplification (CN = 21.4)	CDK12 amplification (CN = 4.0)	CDK12 amplification (CN = 11)	
CDK12			Large genomic rearrangement in CDK12 exon 6 to intron 6 (MAF = 6.87%)	
RAD51C				Copy number loss of exon 4 in RAD51C (CN = 1.2)
FLT4	FLT4 amplification (CN = 4.8)			

MAF, and CN, has been adjusted by the tumor cell proportion when conducting NGS, analysis based on tumor tissue. NGS, next-generation sequencing; MAF, mutation allele frequency; CN, copy number; EGFR, epidermal growth factor receptor; MET, mesenchymal epithelial transition factor; TP53, Tumor Protein P53; ERBB2, Erb-B2, Receptor Tyrosine Kinase 2; CDK12, Cyclin Dependent Kinase 12; RAD51C, RAD51 Paralog C; FLT4, Fms Related Tyrosine Kinase 4.

In May 2023, the patient developed chest tightness and shortness of breath, accompanied by orthopnea and a cough with white frothy sputum. Radiological examinations indicated significant progression in the primary pulmonary, while the chest CT revealed a large pericardial effusion and pleural effusion. Malignant cancer cells were also detected in both the pericardial effusion and pleural effusion. Progression disease was therefore considered, with a progression-free survival of 7.8 months on first-line afatinib treatment. Next-generation sequencing on the pericardial effusion using a 520-genes panel (Burning Rock Biotech, Guangzhou, China) revealed EGFR L858R mutation (mutation allele frequency in pericardial effusion: 30.2%) accompanied by MET amplification (copy number in pericardial effusion: 4.8), CDK12 amplification (copy number in pericardial effusion: 11), ERBB2 amplification (copy number in pericardial effusion: 11). On 17 May 2023, the patient received the regimen of afatinib (30 mg oral once daily) combined with crizotinib (250 mg oral once daily) as the second-line therapy. Follow-up radiological evaluations indicated stable disease. The pericardial effusion and pleural effusion were disappeared during the treatment of afatinib plus crizotinib.

On 2 July 2024, the radiological evaluation indicated the progression of brain lesions, with a progression-free survival of 13.5 months on second-line therapy (afatinib plus crizotinib). On 4 July 2024, the patient was treated with aumolertinib (110 mg oral once daily) and gumarontinib (300 mg oral once daily). On 27^th^ September 2024, the contrast-enhanced brain MRI showed that the brain metastasis was disappeared. This patient is currently continuing follow-up and treatment on aumolertinib and gumarontinib, with a progression-free survival of 3.7 months on this third-line therapy. During targeted therapy with the combination of aumolertinib and gumarontinib, the patient only experienced grade-1 diarrhea, which was relieved after treatment with antidiarrheal medication.

## Discussion

EGFR-TKIs represent the cornerstone of first-line therapy among metastatic NSCLC patients with sensitizing EGFR mutation. Despite the initial efficacy, almost all patients would eventually develop acquired resistance to these targeted therapies. Afatinib is the second-generation EGFR-TKI, which are irreversible inhibiters that covalently bind to EGFR. Previous investigation reported that approximately 60% of EGFR-mutated NSCLC patients with acquired resistance to the first- or second-generation EGFR-TKIs would develop a new mutation within the drug target (Westover et al., 2018). Out of these secondary EGFR mutations, EGFR T790M mutation was the most common mechanism, being present in 40%–55% of the cases. T790M mutation has been shown to alter EGFR-TKI binding and enzymatic activity of EGFR, induced the acquired resistance (Yun et al., 2008). Less common mechanisms of acquired resistance for the first-/second-generation EGFR-TKI include MET amplification, ERBB2 amplification, transformation to small-cell lung cancer, and others. As for third-generation EGFR-TKI, MET amplification was the most frequent acquired resistance mechanism, with the incidence ranged from 7% to 15% in the first-line setting and 5%–50% in the second-line setting (Leonetti et al., 2019). Unlike third-generation EGFR-TKI, MET amplification is less common, which has been only reported in less than 5% of EGFR-mutated NSCLC patients with acquired resistance to first- and second-generation EGFR TKIs. To gain a more comprehensive and in-depth understanding of the acquired resistance mechanism for individual EGFR-mutated NSCLC patients, NGS detection is widely used in clinical practice. Dynamic NGS detection is necessary to identify the accurate resistance mechanism. In this case, the second NGS analysis showed that this patient developed MET amplification after afatinib treatment.

Currently, FISH remains the gold-standard DNA-based method for detection of MET amplification (Duncan et al., 2019). Next-generation sequencing is alternative option, especially when tissue samples are unavailable. Although liquid biopsy using NGS showed high specificity for MET amplification, the limited sensitivity makes it necessary to confirm the negative results through tissue biopsy FISH. Previous analysis showed that a more aggressive and active disease state with high tumor burden often could improve the positive detected rate of MET amplification by liquid biopsy, because this assay relies on sufficient levels of circulating tumor DNA (ctDNA) shed by tumors (Mazieres et al., 2023). For example, in INSIGHT 2 study, the median sum of target lesions for patients with MET amplification identified by liquid biopsy NGS were larger than those identified by tissue biopsy FISH (Wu et al., 2024). In this case, pericardial effusion was examined by NGS assay after the confirmation of malignant pericardial effusion, which detected MET amplification. In contrast, a simultaneous liquid biopsy of peripheral blood failed to detect MET amplification, underscoring the inherent limitations of liquid biopsy on the potential for false-negative results. The high-sensitivity on MET amplification detection using pericardial effusion would be attributed to the higher concentration of metastatic tumor cells within the effusion, offering greater diagnostic reliability compared to peripheral blood-based testing.

Traditional platinum-based doublet chemotherapy is also recommended as subsequent systemic therapy following progression to EGFR-TKIs. Previous trials showed that chemotherapy could provide an ORR of 27%–42.9%, with a mPFS of 4.4–5.6 months for these EGFR-TKI resistant NSCLC patients (Liam et al., 2023; Soria et al., 2015; Yang et al., 2024). Treatment strategies for post-EGFR-TKI therapy in EGFR-mutant NSCLC remain an ongoing challenge. Multiple chemotherapy combination regimens have been attempted to improve the treatment benefits for EGFR-resistant metastatic EGFR-mutated NSCLC, while the role of immunotherapy is uncertain. The ORIENT-31 trial demonstrated that the combination of immunotherapy with chemotherapy significantly improved the clinical outcomes compared with chemotherapy alone among previously treated EGFR-mutation NSCLC (Lu et al., 2022). Conversely, both KEYNOTE-789 and CHECKMATE-722 did not reach the predetermined statistical benchmarks for PFS and OS (Mok et al., 2024; Yang et al., 2023). To improve the clinical benefit for EGFR-TKI resistant NSCLC patients, anti-Vascular Endothelial Growth Factor (VEGF) agent was attempted to combine with immunotherapy based on the finding about the synergistic anti-tumor effect with PD-1 inhibitor (Hegde et al., 2018). Initial subgroup analysis of IMpower-150 trial indicated potential PFS and OS benefits from triple therapy compared to bevacizumab plus chemotherapy among EGFR mutation NSCLC patients (Nogami et al., 2022). Subsequently, trials such as IMpower-151, ORIENT-31, ATTLAS, APPLE, and HARMONI-A were initiated to assess the efficacy of a quadruple regimen involving anti-VEGF, anti-PD-1, and platinum-based doublet chemotherapy for TKI-resistant NSCLC (Lu et al., 2022; Zhou et al., 2023; Park et al., 2024; Shiraishi et al., 2024; Fang et al., 2024). The effectiveness of this combination remains debated, as the IMpower-151 study reported negative outcomes (Zhou et al., 2023), whereas ATTLAS, HARMONI-A, and ORIENT-31 revealed positive results (Lu et al., 2022; Park et al., 2024; Shiraishi et al., 2024; Fang et al., 2024). Thus, the optimal regimen for EGFR-TKI resistant NSCLC patients remained unclear.

Although there is still a lack of consensus on standard of care for EGFR-TKI resistant NSCLC patients who developed MET amplification after the failure of EGFR-TKI due to the absence of robust evidences, the reciprocal crosstalk between EGFR and MET suggests that simultaneous inhibition of these two targets would enhance the clinical benefit of patients with concurrent EGFR and MET aberrations. Additionally, patients with concurrent EGFR mutations and MET amplification following progression on EGFR-TKIs have shown limited progression-free and overall survival when treated with MET-TKI monotherapy. A recent real-world retrospective study showed that patients receiving EGFR-TKI plus MET-TKI have much longer PFS than MET-TKI alone, or chemotherapy (5.0 months vs. 2.3 months, vs. 2.9 months) (Liu et al., 2021). Accumulative evidence has illustrated the clinical benefit of EGFR inhibitor and MET inhibitor, such as crizotinib, capmatinib and savolitinb. Trial likes ORCHARD, SAVANNAH, TATTON showed savolitinib plus osimertinib could provide an ORR of 32%–67% in EGFR-TKI resistant NSCLC harboring MET upregulation including MET amplification and MET overexpression (Wu et al., 2024; Hartmaier et al., 2023). Recently, INSIGHT2 trial showed that tepotinib plus osimertinib has a promising activity and acceptable safety in EGFR-mutated NSCLC patients with MET amplification as a mechanism of resistance to first-line osimertinib (Wu et al., 2024). It has not been characterized well about the resistance mechanisms to combination therapies of EGFR-TKI and MET-TKIs. A retrospective study of 17 patients who developed MET resistance after treatment with EGFR-TKI plus MET-TKI revealed that 15 (88·2%) of 17 patients exhibited loss of MET amplification (Patil et al., 2024). Additionally, MET on-target, bypass signaling pathways were also identified as potential resistance mechanisms. INSIGHT 2 trial showed that 34% of cases developed on-target resistance mutations in EGFR or MET after the progression on tepotinib plus osimertinib (Wu et al., 2024). Among these, 10% of patients have concurrent resistance mutations in both EGFR and MET, underscoring the complexity of resistance mechanisms. In addition, loss of MET amplification, EGFR amplification as well as alterations in other signaling pathways were related with the resistance to the combination of tepotinib and osimertinib. This diversity in resistance mechanisms highlights the necessity for comprehensive molecular testing and the potential subsequent treatment strategies. In this case, afatinib plus crizotinib provided almost a median PFS of 13.5 months, which is much longer than the reported mPFS in previous trials, suggesting this combination regimen may be an alternative treatment for EGFR-TKI resistant NSCLC patients with MET amplification. However, the efficacy of this combination regimen for brain metastases is limited, the patients develop brain metastases after 13.5 months treatment using afatinib plus crizotinib.

Brain metastases are a common complication in EGFR-mutated NSCLC patients, which could be targeted by the third-generation EGFR-TKI as a CNS-penetrant drug (Gillespie et al., 2023). At the point of progression on third-generation EGFR-TKI, brain metastases could be observed among 34%–37% patients with MET amplification (Wu et al., 2024). Thus, the selection of subsequent treatment with CNS-penetrant MET inhibitor would prioritize the need for effective intracranial control. For instance, the combination of tepotinib with osimertinib has demonstrated promising clinical efficacy in patients with CNS metastases, yielding an intracranial objective response rate of 29.2%, an intracranial disease control rate of 79.2%, and a median intracranial progression-free survival of 7.8 months (Wu et al., 2024). These findings underscore the importance of considering intracranial outcomes when devising treatment strategies for this patient population. This case also further demonstrated the clinical significance of CNS-penetrant targeted drugs in this clinical setting. As CNS-penetrant targeted drugs, both aumolertinib and gumarontinib showed the significant intracranial anti-cancer activity when the patients developed brain metastases.

Of course, the inherent limitations of case studies should be noticed, including the small sample size and lack of generalizability, etc. The potential variability in treatment response across diverse patient populations with different genetic and environmental backgrounds would also be considered, especially among these EGFR-mutated NSCLC patients. The impact of mutation subtypes and co-mutations on the therapeutic efficacy of this regimen remains unclear. Therefore, this approach required the further validation in larger, multi-center clinical trials.

## Conclusion

In conclusion, although MET amplification is the most frequent resistance mechanism to first-line osimertinib, which is also an important resistance mechanism to second-generation EGFR-TKIs. After resistance to EGFR-TKI combined with crizotinib, other novel MET inhibitors combined with EGFR-TKIs can be considered. The combination of aumolertinib plus gumarontinib may offer a promising strategy to overcome MET amplification-induced EGFR-TKI resistance. This case underscores the importance of re-challenge using third-generation EGFR-TKI with novel MET-TKI after the failure of second-generation EGFR-TKI plus crizotinib in EGFR-TKI resistant NSCLC patients with MET amplification, especially in patients with brain metastases. The successful application of aumolertinib plus gumarontinib highlights its potential in overcoming MET amplification-induced EGFR-TKI resistance, which warrants further investigation in future large-scale clinical trials due to the inherent limitations of case studies.

## Data Availability

The raw data supporting the conclusions of this article will be made available by the authors, without undue reservation.
